# Effects of cessation of cigarette smoking on eicosanoid biomarkers of inflammation and oxidative damage

**DOI:** 10.1371/journal.pone.0218386

**Published:** 2019-06-28

**Authors:** Joseph P. McElroy, Steven G. Carmella, Alisa K. Heskin, Mei Kuen Tang, Sharon E. Murphy, Sarah A. Reisinger, Joni A. Jensen, Dorothy K. Hatsukami, Stephen S. Hecht, Peter G. Shields

**Affiliations:** 1 Center for Biostatistics, Department of Biomedical Informatics, The Ohio State University, Columbus, OH, United States of America; 2 Masonic Cancer Center, University of Minnesota, Minneapolis, MN, United States of America; 3 The Ohio State University Comprehensive Cancer Center, Columbus, OH, United States of America; Virginia Commonwealth University School of Pharmacy, UNITED STATES

## Abstract

The urinary metabolites “prostaglandin E_2_ metabolite” (PGE-M) and (*Z*)-7-[1*R*,2*R*,3*R*,5*S*)-3,5-dihydroxy-2-[(*E*,3*S*)-3-hydroxyoct-1-enyl]cyclopentyl]hept-5-enoic acid (8-*iso*-PGF_2α_) are biomarkers of inflammation and oxidative damage, respectively, and are elevated in cigarette smokers. Relatively little is known about the effects of smoking cessation on these biomarkers. To investigate this, current cigarette smokers interested in quitting were recruited and invited to participate in a smoking cessation study where varenicline (Chantix) and brief supportive behavioral counseling were offered at each visit after baseline. Subjects returned to the clinic during the 12 week treatment phase for 9 visits post cessation on days 3, 7, 14, 21, 28, 42, 56, 70 and 84. Urine samples were collected at each visit and analyzed by liquid chromatography-tandem mass spectrometry for PGE-M, 8-*iso*-PGF_2α_, and cotinine. Cotinine values demonstrated that 15 of 38 subjects quit smoking for the entire 84 day period. Significant decreases in mean levels of PGE-M and 8-*iso*-PGF_2α_ per milligram creatinine were observed in these subjects, by 44% (p = 0.0014) and 27% (p<0.001), respectively. The results of this study demonstrate that cessation of smoking for 84 days results in modest but significant declines in urinary PGE-M and 8-*iso*-PGF_2α_ indicating reductions in systemic inflammation and oxidative damage. Given that levels were only modestly decreased, these markers are not specific to tobacco-smoke exposure. The modest declines in these biomarkers should be considered when planning studies with ex-smokers. There is a “hangover” from smoking that lasts at least 3 months.

## Introduction

**Fig 1:**
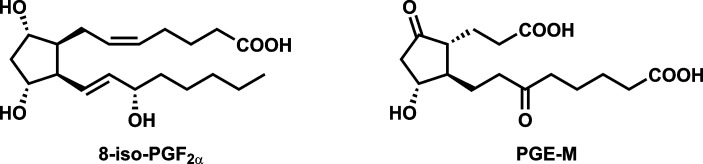
Structures of 8-*iso*-PGF_2α_ and PGE-M.

Inflammation and oxidative damage, which are closely related and interlinked phenomena, are significant factors in diseases caused by cigarette smoking including cancer, cardiovascular disease, and chronic obstructive pulmonary disease.[1, 2] The urinary metabolites “prostaglandin E_2_ metabolite” (PGE-M) and (*Z*)-7-[1*R*,2*R*,3*R*,5*S*)-3,5-dihydroxy-2-[(*E*,3*S*)-3-hydroxyoct-1-enyl]cyclopentyl]hept-5-enoic acid (8-*iso*-PGF_2α_) are accepted biomarkers of inflammation and oxidative damage, respectively, and are part of a group of measures that have been termed “biomarkers of potential harm”.[3–6] Fig 1.

PGE-M is a metabolite of prostaglandin E_2_, the product of the immediate-early response gene prostaglandin G/H synthase-2 (COX-2), which is induced at sites of inflammation. 8-*iso*-PGF_2α_ is one of the F_2_-isoprostanes formed during the non-enzymatic free radical initiated oxidation of arachidonic acid, a polyunsaturated fatty acid which is ubiquitous in the human body. Multiple investigations have reported that urinary levels of PGE-M and 8-*iso*-PGF_2α_ are elevated in smokers compared to non-smokers.[4, 7–13] Several previous studies have examined the effects of smoking cessation on levels of PGE-M and 8-*iso*-PGF_2α_,[14–19] with somewhat diverse results.

We aimed to determine whether significant decreases in levels of these biomarkers would be observed after 12 weeks of abstinence from cigarette smoking, as confirmed by urinary cotinine measurements. The results of this study could be critical for the design of subsequent investigations to determine the effects on inflammation and oxidative damage of switching from cigarette smoking to other forms of nicotine use, such as smokeless tobacco or e-cigarettes. Therefore, in the study reported here, cigarette smokers used varenicline (Chantix) as a smoking cessation aid, and visited our clinic regularly over a 12-week period to provide urine samples which were analyzed for PGE-M, 8-*iso*-PGF_2α_ and cotinine by validated liquid chromatography-tandem mass spectrometry methods.

## Materials and methods

### Clinical study

This study was conducted in Washington, DC and Minneapolis, MN. The study was approved by the Georgetown University Institutional Review Board (2007–322) and the University of Minnesota Institutional Review Board (0705M08181), and 83 subjects provided written consent. It had a within-subject design comparing several biomarkers of tobacco-related toxicants during two baseline weeks of smoking and at nine time points during 12 weeks of cessation. Current cigarette smokers interested in quitting were recruited from prior studies; local print, radio and internet advertisements; and snowball sampling. Respondents were invited to participate in the 14 week smoking cessation study where varenicline (Chantix) and brief supportive behavioral counseling were offered at each visit after baseline. Eligible participants included adults (age 18–65 years) who smoked an average of 10 cigarettes/day for at least one year. Participants had to be in good physical and mental health as evidenced by a medical history with no unstable medical conditions, and physician approval. Participants were excluded if they had poor or unstable physical or mental health per clinician’s clinical judgment; immune system disorders affecting biomarkers (as determined by the Principal Investigator), included but not limited to rheumatologic illness, lupus, and multiple sclerosis; contraindications to varenicline, such as kidney disease, pregnancy, or lactating; unstable psychiatric symptoms (currently on psychiatric medications or experiencing symptoms of depression, anxiety, or other psychiatric disorders); recent past history of depression (use of medications and/or any depressive/anxiety symptoms in < 1 year); current use of psychotropic medications; current (within 3 months) alcohol or drug abuse; regular use of other tobacco, cigars, smokeless tobacco or nicotine replacement products within the last month or during the research study; chronic use of any drug that could interact with the study drug or biomarker measures (determined by the site medical provider or principal investigator); or insulin dependent diabetes. Subjects visited the clinic for an orientation visit and then 2 baseline visits that occurred approximately one week apart. Subjects returned to the clinic during the 12 week treatment phase for 9 interval visits, at post cessation on days 3, 7, 14, 21, 28, 42, 56, 70 and 84. A 24h urine sample from the second baseline visit and each of the interval visits was analyzed. They were instructed to quit 7 days after the second baseline visit. Visits were conducted throughout the day. Subjects were instructed to start the urine collection on the day before the scheduled visit. They began collection with the second void in the morning and continued to collect all urine throughout the day and night through the first void on the second day (day of the visit). Subjects were called to remind them to start the 24 hr urine collection the day before their next visit and bring the sample to the visit. They were offered varenicline and instructed to begin taking one 0.5 mg tablet the day after the second baseline visit and also for the next 2 days. The dosage was increased to 0.5 mg twice daily for the next 4 days before the quit day. From the quit day throughout the 12 treatment weeks, the dosage of the study drug was 1 mg twice daily. NicAlert strips and CO <6 were used at the visits to confirm abstinence. Subjects received medication and supportive counseling at no cost while participating in the study. Contingency management, providing subjects monetary reinforcements for being abstinent, was used to increase retention and abstinence. Starting at the first smoke-free visit, compensation escalated by $10 at each subsequent visit for subjects with confirmed smoke-free status. Visit compensation reverted to $0 if subjects were not abstinent. Subjects could earn up to $605 for the entire study, including $5 travel for each visit and $100 bonus for successfully completing the study smoke-free.

### Analysis of PGE-M

This was adapted from Neale and Dean.[20] For each sample, a 400 μL aliquot of urine was transferred to a well in a 2 mL V-bottom 96-well collection plate (Analytical Sales and Services, Cat. # 59623–23) and was spiked with 120 pmol PGEM-d_6_ (Cayman Chemical, Ann Harbor, MI) dissolved in 10% aqueous formic acid and 40 μL of formic acid (ACS grade ≥98%). The plates were capped with a sealing mat (Phenomenex, Cat. # AH0-8597), vortexed at 2000 rpm for 5 sec and heated for 14–16 h at 60°C to convert PGE-M to PGA-M by dehydration. Each sample plate also contained 3 positive and 2 negative controls as pooled smokers’ (n = 9) urine and H_2_O, respectively. Bond Elut C18 96 square-well plates (100 mg) from Agilent (Cat. # A396011C) were used for solid phase extraction. The plates were processed on a CEREX System 96 processer. The plate was conditioned sequentially with 1 mL of MeOH, 1 mL of CH_3_CN and 3 mL of 50 mM potassium phosphate buffer, pH 3. Following application of the samples, the plate was washed with additional volumes of phosphate buffer and hexanes, 3 mL each, before elution with 1.25 mL ethyl acetate into a Tru-Taper 96-well collection plate (Analytical Sales and Services, Cat. # 968820). The ethyl acetate was evaporated to dryness using a Speedvac concentrator (Thermo Fisher), and the plates were capped and stored in a -20°C freezer until LC-MS analysis. For injection, samples were reconstituted in 50 μL of 5% aqueous CH_3_CN with 0.1% formic acid.

Ten μL of the reconstituted sample was injected onto the LC-MS/MS system for SRM analysis on a TSQ Quantum Discovery Max instrument (Thermo Fisher Scientific, Waltham, MA) operated in the negative ion APCI mode. Chromatography was performed on an Agilent 1100 HPLC system (Agilent Technologies, Inc., Santa Clara, CA) with a 50 x 3.0 mm, 2.6 μm Kinetex C18 LC column (Phenomenex, Torrance, CA). Mobile phase A was 15 mM ammonium acetate and solvent B was CH_3_OH. The LC column temperature was 45°C and the flow rate was 0.34 mL/min. The column gradient was at 98% A and 2% B for 2 min, then ramped up to 12% B over 3 min and held there for 2 min. Then, solvent B was increased to 95% within 0.5 min and held for 1 min. Finally, the column was re-equilibrated for 4 min at initial conditions. The MRM transitions monitored were the following: PGA-M-d_6_, *m/z* 315 → *m/z* 297 (quantifier) and *m/z* 315 → 149 (qualifier); PGA-M, *m/z* 309 → *m/z* 291 (quantifier) and *m/z* 309 → 143 (qualifier). Other MS/MS parameters were as follows: collision energy for transitions; 15 V for *m/z* 315 → *m/z* 297, *m/z* 309 → *m/z* 291 and 23 V for *m/z* 315 → 149, *m/z* 309 → 143; Q1 = 0.7 FWHM and Q3 = 0.7 FWHM; scan width *m/z* = 0.10; scan time 0.25 s; vaporizer temperature 80°C; capillary temperature 270°C; N_2_ sheath gas pressure, 60 psi; N_2_ auxiliary gas pressure, 5 psi. Argon was the collision gas. Quantitation was performed as described for 8-*iso*-PGF_2α_. Inter-assay precision was 5.6%, intra-assay precision was 3.5% (CV), average accuracy was 102%, and the limit of detection was 1.1 pmol/ml urine. A typical LC-MS/MS trace of PGE-M is provided in S1 Fig.

### Analysis of 8-*iso*-PGF_2α_

The method was adapted from Yan *et al*.[10] 8-*iso*-PGF_2α_ and 8-*iso*-PGF_2α_-d_4_ were procured from Cayman Chemical (Ann Arbor, MI). For each sample, a 200 μL aliquot of urine was transferred to a well in a V-bottom 96-well collection plate (Analytical Sales and Services, Cat. # 59623–23). To each sample was added 20 μL of 0.1 ng/μL 8-*iso*-PGF_2α_-d_4_ dissolved in 80% aqueous methanol and 20 μL of formic acid (ACS grade, ≥98%). The plate was covered with a 96-square well sealing mat (Phenomenex, Cat. # AH0-8597) and the samples were vortexed at 2000 rpm for 5 sec. Each sample plate also contained 3 positive and 2 negative controls in the forms of pooled (n = 9) smokers’ urine and H_2_O, respectively. Bond Elut C18 96 square-well plates (100 mg) from Agilent (Cat. # A396011C) were used for solid phase extraction (SPE). The plates were processed on a CEREX System 96 processor. Each plate was conditioned sequentially with 1 mL of MeOH, 1 mL of CH_3_CN, and 3 mL of 50 mM potassium phosphate buffer, pH 3. Following application of the samples, the plate was washed with additional volumes of phosphate buffer and hexanes, 3 mL each, before elution with 1 mL ethyl acetate into a Tru-Taper 96-well plate (Analytical Sales and Services, Cat. # 968820). The ethyl acetate was evaporated to dryness using a Speedvac concentrator (Thermo Fisher), and the plates were capped and stored at -20°C until LC ESI^—^MS/MS analysis. For injection, samples were reconstituted in 30 μL 80:20 H_2_O:MeOH (v/v) with 0.15% NH_4_OH.

Sample analysis was performed using LC ESI^—^MS/MS on a Vantage triple quadrupole mass spectrometer (Thermo Scientific, Pittsburgh PA) using negative electrospray mode,with a Dionex Ultimate 3000 Rapid separation (RSLC) HPLC system (Thermo Scientific, Pittsburgh PA). An XBridge BEH C18, 2.5 μm, 50 mm x 1.0 mm column (Waters Corp Cat. # 186003118) was used. Sample injection volumes were 2 μL. The column was kept at room temperature and H_2_O and 95:5 CH_3_CN:MeOH (v/v) both containing 0.15% NH_4_OH were the eluting solvents. A shallow elution gradient with a flow rate of 45 μL/min was used to achieve separation of 8-*iso*-PGF_2α_ from its isomers; only 8-*iso*-PGF_2α_ was quantified. After the initial condition of 5% organic, the percentage was increased to 12% over 0.5 min, held at 12% for 2 min, increased to 16% over 3 min, and held at 16% for 2 min before increasing to 95% organic over 4 min, holding for 2 min, and re-equilibrating at initial conditions for 2.5 min. The MRM transitions monitored were: 8-*iso*-PGF_2α_, *m/z* 353 → *m/z* 193 (quantifier) and *m/z* 353 → *m/z* 173 (qualifier); 8-*iso*-PGF_2α_-d_4_, *m/z* 357 → 197 (quantifier) and 357 → 177 (qualifier). Fragmentation was achieved with a collision energy of 25 V and gas pressure of 1.2 mTorr. Argon was the collision gas. Quadrupole resolution was set at *m/*z 0.7 for both Q1 and Q3, with a scan width of *m/z* 0.1 and scan time of 0.25 s. The capillary temperature was set to 255°C, N_2_ sheath gas pressure at 40, and N_2_ auxiliary gas pressure at 2 psi. Peak areas were quantified using Thermo Xcaliber Version 2.2 Quan browser. Peak areas were converted to amounts using a linear standard curve comparing area ratios to amount ratios of varying amounts of the analyte and a constant amount of the internal standard, R^2^ = 0.998.Inter-assay precision was 7.0%, intra-assay precision was 2.9% (CV), average accuracy was 101%, average recovery was 65%, and the limit of detection was 0.03 pmol/ml urine. A typical LC-MS/MS trace of 8-*iso*-PGF_2α_ is provided in S1 Fig.

### Analysis of cotinine

This was performed by LC-MS/MS as described previously.[21] The LOD was 1.2 pmol/ml urine.

### Analysis of creatinine

Creatinine was analyzed using a colorimetric microplate assay (CRE34-K01) purchased from Eagle Bioscience. Urinary creatinine reacts with picric acid under alkaline conditions to produce an orange color which is quantified by absorption spectroscopy near 500 nm wavelength. This reaction also occurs non-specifically with other components of urine. The difference in color intensity before and after the addition of acid is a direct estimate of creatinine concentration in the sample.

### Statistical methods

Data were available for 15 subjects after removal of 12 subjects because they were later determined to be non-smokers, very light smokers, or did not stop smoking during the study. For instances where PGE-M, 8-*iso*-PGF_2α_ (pmol/mL urine), or cotinine (ng/mL) were below the limit of detection (LOD), values were assigned 0.5*LOD. PGE-M and 8-*iso*-PGF_2α_ data were normalized by dividing by mg creatinine to account for differences in urine concentration and log transformed because of right skewedness. Metabolite data for testing the associations with demographic variables were generated by taking the residuals from a linear model with the latest time point with complete data (day 56) as the dependent variable and baseline measure as the independent variable (to adjust the day 56 values for differences at baseline). Associations between sex and race with the metabolites were tested with a two sample Welch’s t-test, and association between age and the metabolites was tested with a linear model. For association between PGE-M or 8-*iso*-PGF_2α_ across samples and times, residuals from a linear model with PGE-M or 8-*iso*-PGF_2α_ as dependent variables and subject ID as the independent variable (to remove overall subject effects) were correlated. To analyze the trend in PGE-M and 8-*iso*-PGF_2α_ levels over time after quitting, linear mixed models were employed with PGE-M or 8-*iso*-PGF_2α_ as the dependent variables, a random effect for patient, covariables for baseline measure, cotinine, and gender, and a main effect of time after quitting. Paired t-tests were employed as follow-up to compare specific pairs of time points. Because one subject had unusually high PGE-M levels (possibly from a medical condition; this was the only subject to take Tegretol), analyses were also performed after removing this questionable sample to insure that conclusions were not dependent on these data. All analyses were performed in R (version 3.4.4).[22] Comparison-wise p-values < 0.05 were considered significant.

## Results

Data from 15 subjects were analyzed after exclusion of non-smokers, very light smokers, or subjects who did not stop smoking based on inspection of the decrease in urinary cotinine over the course of the study, typically to less than 15 ng/ml (Table 1). There were 8 African Americans and 7 Whites (Caucasians). Ten subjects were female. Two PGE-M values, no 8-*iso*-PGF_2α_ values, and 22 cotinine values were below the LOD. Race, gender, and age were not significantly associated with baseline PGE-M or 8-*iso*-PGF_2α._ There was only one report of anti-inflammatory drug use, on day 21 of the study by one subject. Sex and age were not associated with either day 56 residual metabolite (p > 0.05), and race was associated with only PGE-M (p = 0.01). The correlation between PGE-M or 8-*iso*-PGF_2α_ across samples and times was 0.46 (p < 0.001).

**Table 1:** Demographic and baseline data for PGE-M and 8-*iso*-PGF_2α_.

Variable	Level	Overall
n		15
Gender (%)	*F*	10 (66.7)
	*M*	5 (33.3)
Race (%)	*African American*	8 (53.3)
	*Caucasian*	7 (46.7)
Age (mean (sd)), years		44.9 (11.7)
Baseline_PGE-M (mean (sd)), log_10_ pmol/mg creatinine		1.75 (0.36)
Baseline_8-*iso*-PGF_2α_ (mean (sd)) log_10_ pmol/mg creatinine		0.00 (0.15)
Baseline cotinine (mean (sd)) ng/mg creatinine		2912 (714.6)

### PGE-M

**Fig 2:**
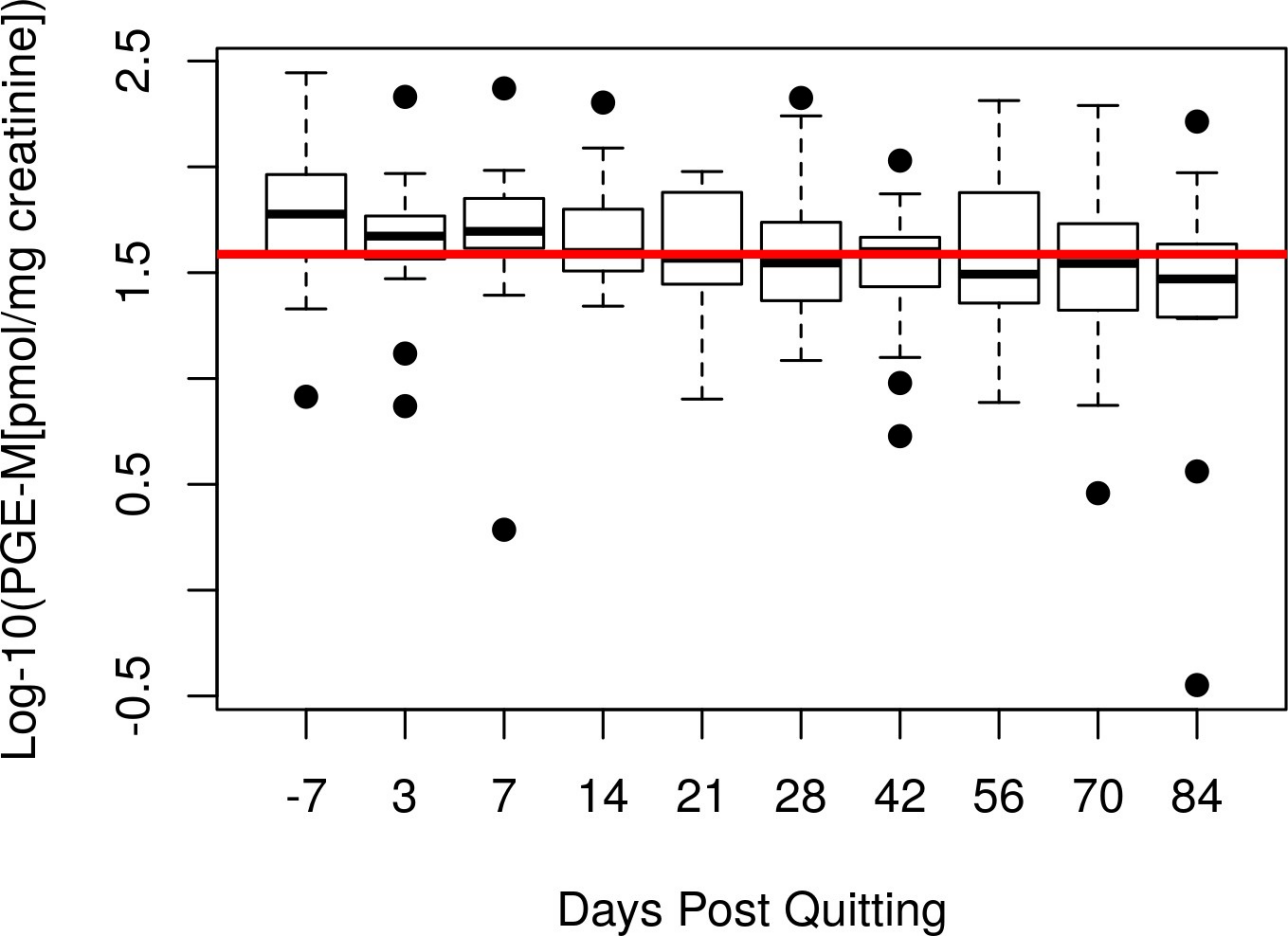
Boxplot of Log-10 PGE-M vs Time Post Quitting. The red line is the overall mean. Values on the original scale were 74.8 pmol/mg creatinine at baseline and 43.1 pmol/mg creatinine at day 84.

**Fig 3:**
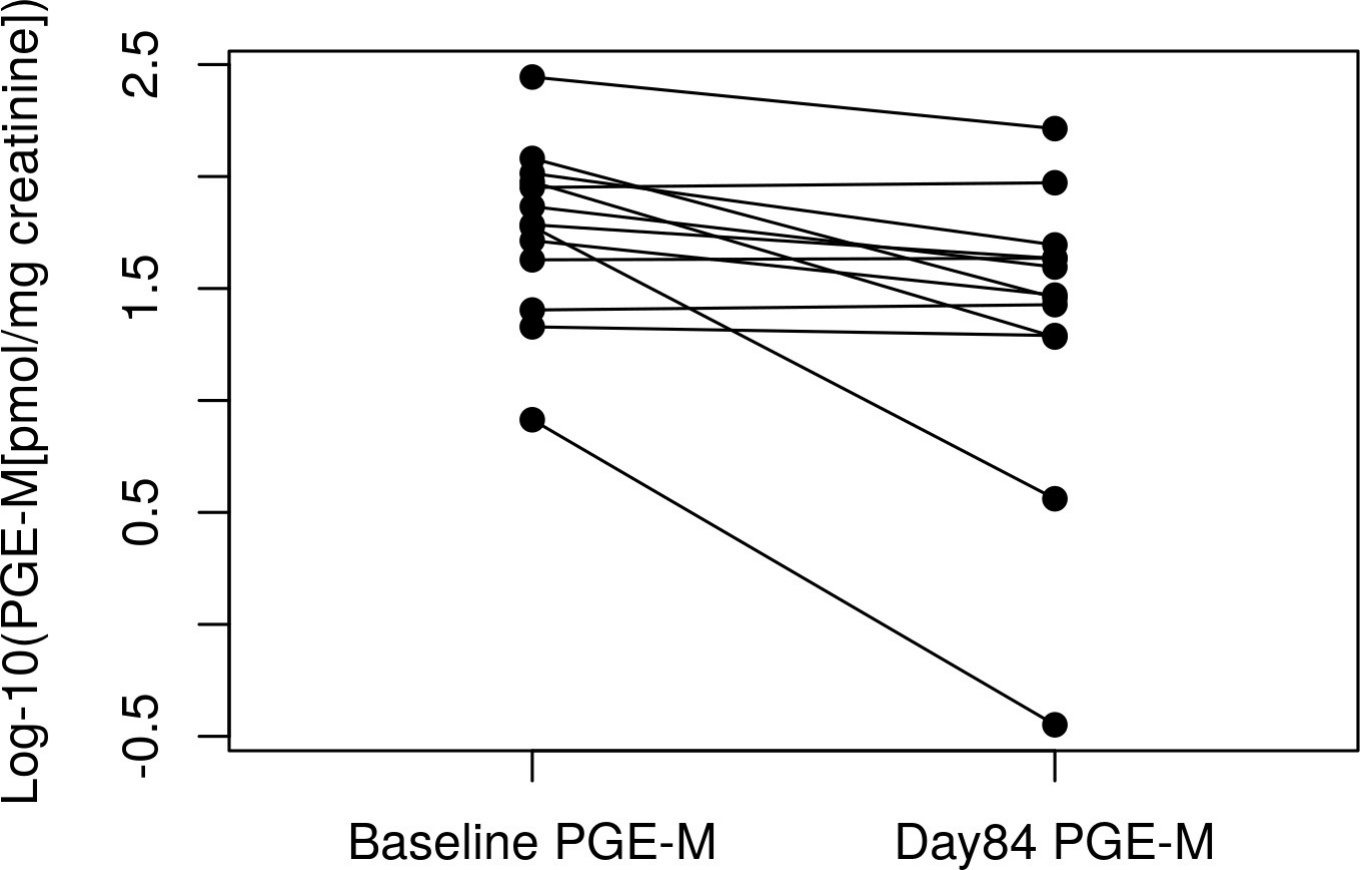
Ladder plot of baseline PGE-M vs. Day 84 PGE-M. Two individuals were missing day 84 PGE-M data; therefore n = 13. Paired t-test p-value = 0.009.

Using a multivariable linear mixed model, time was significantly associated (p = 0.0014) with a decrease in PGE-M levels (S1 Table; Fig 2). The estimate for “time” is for a one day change in log_10_ PGE-M space. This is equivalent to a 44% decrease in mean PGE-M on the original scale over the course of the study (84 days). Baseline log_10_ PGE-M was also significant in this multivariable model (p < 0.001), but race and sex were not (p > 0.05). Time was still significant (p = 0.001) after removing data from one subject with extremely high PGE-M and 8-epi-PGF_2α_ values, as well as after removing the sample with anti-inflammatory drug use (p < 0.001). We also compared baseline vs. day 3 and baseline vs. day 84 with paired t-tests to examine the immediate and longer term effects of quitting. Baseline vs. day 3 was non-significant (p = 0.14); baseline vs. day 84 was significant (p = 0.009; Fig 3). Pairwise comparisons of baseline PGE-M vs. each time point are provided in S2 Table.

### 8-*iso*-PGF_2α_

**Fig 4:**
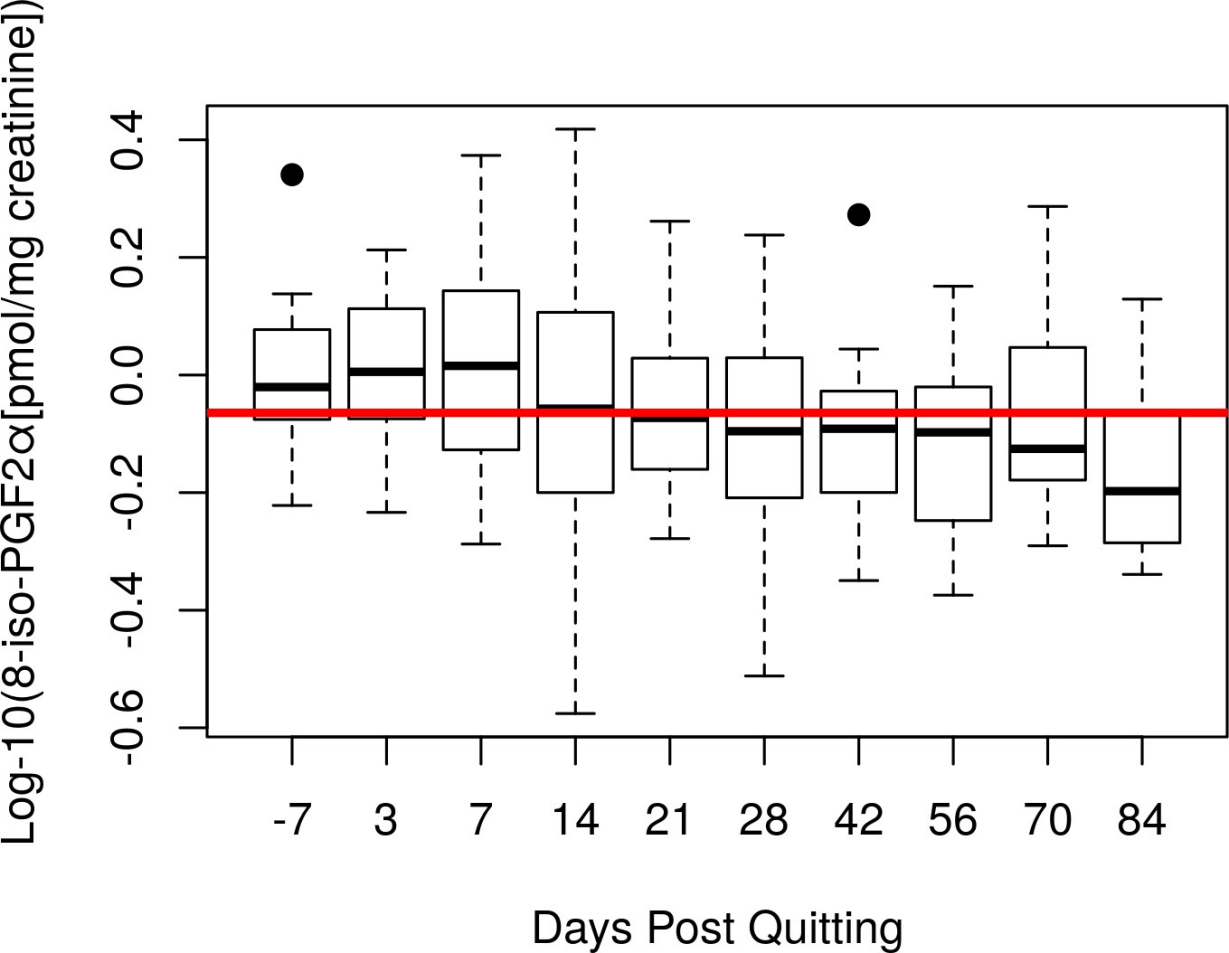
Boxplot of Log-10 8-*iso*-PGF_2α_ vs Time Post Quitting. The red line is the overall mean. Values on the original scale were 1.05 pmol/mg creatinine at baseline and 0.73 pmol/mg creatinine at day 84.

**Fig 5:**
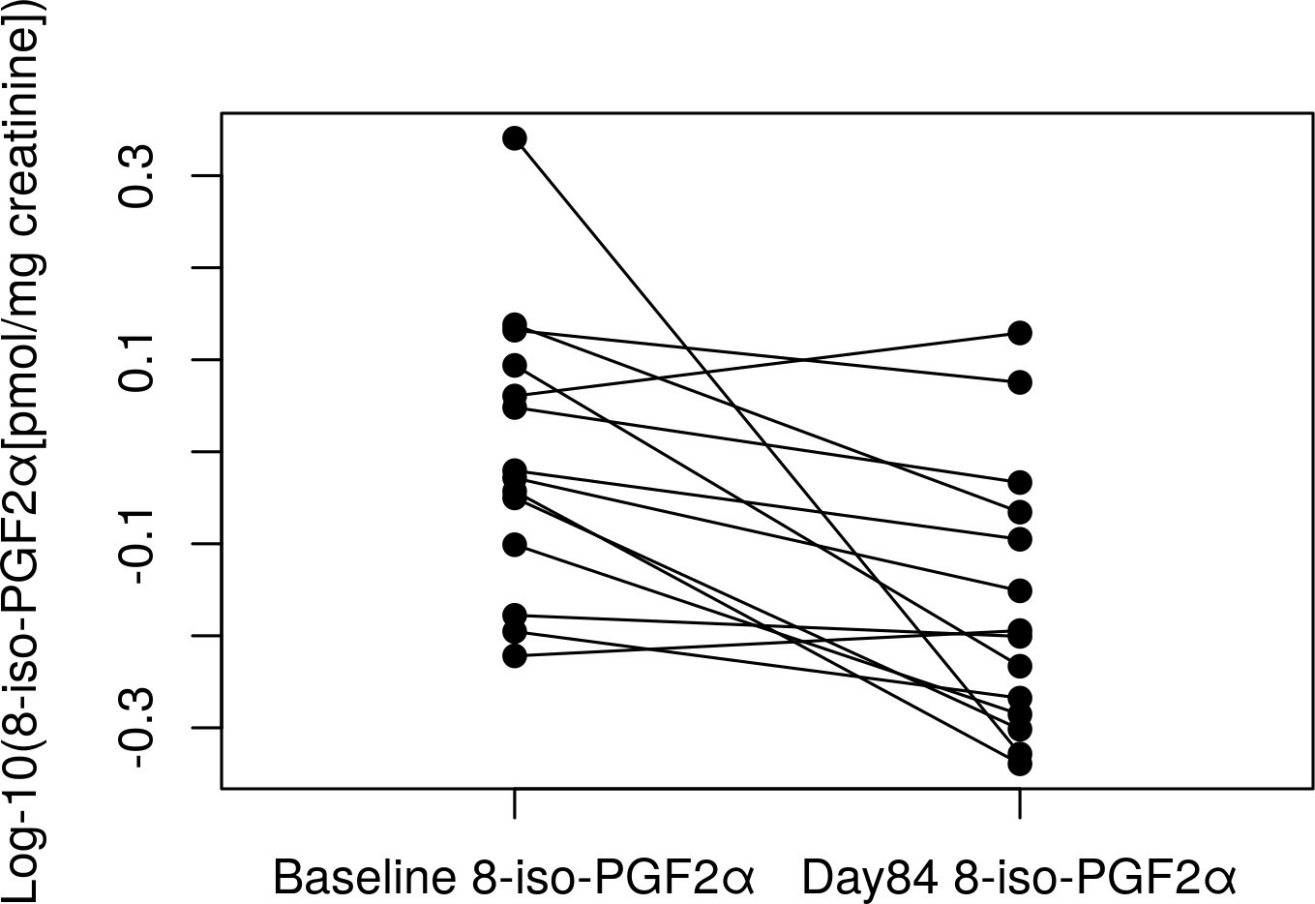
Ladder plot of baseline 8-*iso*-PGF_2α_ vs. Day 84 8-*iso*-PGF_2α_. One individual did not have a day 84 sample; therefore n = 14. Paired t-test p-value = 0.007.

Using a multivariable linear mixed model, time was significantly (p<0.001) associated with a decrease in 8-*iso*-PGF_2α_ levels (S3 Table; Fig 4). The estimate for “time” is for a one day change in log-10 8-*iso*-PGF_2α_ space. This is equivalent to a 27% decrease in mean 8-*iso*-PGF_2α_ on the original scale over the course of the study. Baseline log 10 8-*iso*-PGF_2α_ and race were also significant in this multivariable model (p < 0.05), but sex was not (p > 0.05). Time was still significant (p <0.001) after removing data from one subject with extremely high PGE-M and 8- *iso*-PGF_2α_ values,as well as after removing the sample with anti-inflammatory drug use (p = 0.0001). We also compared baseline vs. day 3 and baseline vs. day 84 with paired t-tests to examine the immediate and longer term effects of quitting. Baseline vs. day 3 was non-significant (p = 1.00); baseline vs. day 84 was significant (p = 0.007; Fig 5). Pairwise comparisons of baseline 8-*iso*-PGF_2α_ vs. each time point are provided in S4 Table.

## Discussion

We analyzed the “biomarkers of potential harm” PGE-M and 8-*iso*-PGF_2α_ in urine samples from 15 cigarette smokers who used varenicline as an aid to quit smoking. Measurements were made at baseline and 9 time points over the course of their cessation of smoking. Urine cotinine was quantified to confirm abstinence at all time points from day 3 to day 84. We found significant but modest decreases in both PGE-M (44% in 84 days) and 8-*iso*-PGF_2α_ (27%). These decreases are considerably less than observed for typical biomarkers of exposure. For example, we previously reported decreases of 92%, 87%, 88%, 39%, and 65% in biomarkers of exposure to 1,3-butadiene, acrolein, benzene, pyrene, and NNK, respectively, only 3 days after cessation of smoking.[23] These differences clearly reflect the fact that changes in PGE-M and 8-*iso*-PGF_2α_ are dependent on physiological alterations in smokers who stopped while biomarkers of exposure are dependent only on toxicant dose. Factors affecting PGE-M levels include high saturated fat intake, obesity, and poor self-reported health status[3] while 8-*iso*-PGF_2α_ levels are increased by a wide variety of conditions that generate reactive oxygen species including endogenous processes and exposure to UV radiation and environmental toxicants [5]. It will be important to take the modest decreases observed here into account when planning studies on the effects of switching from cigarette smoking to non-combustible forms of tobacco or to e-cigarettes because there is a potential “hangover” from smoking due to some of the factors noted above that can persist into the period of new product use.

Several previous studies have examined the effect of smoking cessation on urinary levels of 8-*iso*-PGF_2α_ and one has also investigated PGE-M. Reilly *et al*. used gas chromatography–mass spectrometry to study 6 male smokers who smoked more than 30 cigarettes per day, then stopped for 3 weeks by using transdermal nicotine as a cessation aid. Urinary cotinine levels declined and then reached a plateau, presumably due to use of transdermal nicotine. A 23% decline in 8-*iso*-PGF_2α_ was observed, but the resulting level was still more than twice as great as the non-smoker levels measured in the same study.[14]

Pilz *et al*. studied 47 people (26 males) who quit smoking (with no pharmaceutical aid) and had no additional risk factors.[16] Using an immunoassay, they observed a rapid decrease in 8-*iso*-PGF_2α_ which reached statistical significance within 3 days of quitting, and decreased by 40% from the baseline value within 2 weeks. Biomarker data to confirm non-smoking status was not presented. A follow up study of 71 patients with clinically manifested atherosclerosis and various risk factors showed similar results.[17]

Mah *et al*. carried out a study of 23 smokers who received nicotine replacement therapy and abstained from smoking for 24h with placebo or oral administration of a gamma-tocopherol rich mixture.[19] 8-*iso*-PGF_2α_ was determined by LC-MS/MS. There was no observed decrease in 8-*iso*-PGF_2α_ in either group.

King *et al*. evaluated the effects of smoking cessation on 8-*iso*-PGF_2α_, determined by LC-MS/MS, in the longitudinal Wisconsin Smokers Health Study-2, which was designed to examine the natural history of smoking and smoking cessation.[15] They found a significant 14% decrease of 8-*iso*-PGF_2α_ in 344 subjects who abstained from smoking for one year.

Goettel *et al*. recently reported a study in which they followed 39 smokers over the course of 3 months of smoking cessation.[18] The subjects were all healthy male smokers who smoked more than 15 cigarettes per day during the past 12 months and were motivated to quit smoking. They attended a series of stationary and ambulatory visits to the clinic as described.[24] Diets were controlled during the stationary visits. No cessation therapy was used. Sampling was conducted at the start of the study and after 1 week, 1 month, and 3 months of smoking cessation. Compliance was monitored by breath CO and urinary and saliva cotinine. They observed a significant decrease of 21% for 8-*iso*-PGF_2α_, but not in PGE-M.

Thus, with the exception of the studies by Pilz *et al*., which did not use LC-MS/MS analysis, and that of Mah *et al*. in which cessation was for only one day, the overall results show a relatively slow and modest, yet significant, decrease in 8-*iso*-PGF_2α_ over the course of 3 weeks to one year, while the only other study to report the effects of cessation on PGE-M found no significant decrease. These results, together with other published data, indicate that the inflammation and oxidative damage produced by cigarette smoking persist and decrease only slowly or not at all upon cessation, and that there are likely other sources of inflammation and oxidative damage.

Our data for the subjects who stopped smoking are comparable to data in the literature for non-smokers. In one study, levels of 8-*iso*-PGF_2α_ in non-smokers averaged 0.83 pmol/mg creatinine compared to a mean of 0.73 pmol/mg creatinine observed here at day 84.[10] For PGE-M, a value of 30.2 pmol/mg creatinine has been reported for non-smokers, compared to 43.1 pmol/mg creatinine which we observed at day 84.[8] These data support the concept that the residual levels of 8-*iso*-PGF_2α_ and PGE-M arise from sources other than the effects of cigarette smoking.

This study has some strengths and limitations. Smoking cessation studies that use within subject comparisons are among the best evidence for validating the use of a biomarker for cigarette smoke exposure and harm, justifying its use for long term studies that assess disease risk, e.g., cohort studies. The trial design allowed for frequent assessments and was of long enough duration to identify the time for smoking-related effects to wash out and reach a plateau. Separately, this study used biochemical data to confirm abstinence, and the use of varenicline rather than nicotine replacement therapy allows for the assessment of quitting all smoke constituents. A limitation is that the results may not be generalizable to the general population of smokers, reflecting only the effects of volunteers in a clinical trial who may not represent other smokers or otherwise alter their behavior during the trial. This study cannot determine why the levels of 8-*iso*-PGF_2α_, and PGE-M remained after quitting, such as what dietary sources or endogenous mechanisms contributed.

In summary, this study has demonstrated significant but modest decreases in levels of the biomarkers of inflammation (PGE-M, 44%) and oxidative damage (8-*iso*-PGF_2α_,27%) over an 84 day period of cigarette smoking cessation, as confirmed by urinary cotinine, in subjects who used the drug varenicline. These results are consistent with those of most other studies, indicating that decreases in these biomarkers of potential harm occur only slowly upon smoking cessation.

## Supporting information

S1 Table Results from PGE-M multivariable mixed linear model. Model: PGE-M = Baseline PGE-M + Sex + Race + Time. Estimates are in units of Log10(pmol PGE-M/mg creatinine). The estimate of the time effect on PGE-M is for a one unit change in time (1 day) on log10 PGE-M. Std.Error = Standard error of the estimate; t value is the t statistic for the estimate.
(DOCX)

S2 Table Results from pairwise comparisons of baseline PGE-M vs. each time point. Paired t-test employed between baseline and each time-point. Geometric fold change is 10^mean of paired differences).
(DOCX)

S3 Table Results from 8-iso-PGF 2α multivariable mixed linear model. Model: 8-iso-PGF 2α = Baseline 8-iso-PGF 2α + Sex + Race + Time. Estimates are in units of Log10 (pmol 8-iso-PGF 2α /mg creatinine). The estimate of the time effect on 8-iso-PGF 2α is for a one unit change in time (1 day) on log10 8-iso-PGF 2α. Std.Error = Standard error of the estimate; t value is the t statistic for the estimate.
(DOCX)

S4 Table Results from pairwise comparisons of baseline 8-*iso*-PGF_2α_ vs. each time point. Paired t-test employed between baseline and each time-point. Geometric fold change is 10^mean of paired differences).
(DOCX)

S1 Fig Typical LC-MS/MS trace of PGE-M (A) and 8-*iso*-PGF_2α_ (B) in a smoker's urine.
(TIFF)

S1 File Data. (XLSX)
