# Supplementary material for: Effects of cessation of cigarette smoking on eicosanoid biomarkers of inflammation and oxidative damage
Source: PLoS One. 2019 Jun 28;14(6):e0218386. doi: 10.1371/journal.pone.0218386 (PMC6599218; doi:10.1371/journal.pone.0218386)
Supplement: S1 Table — Model: PGE-M = Baseline PGE-M + Sex + Race + Time. Estimates are in units of Log10(pmol PGE-M/mg creatinine). The estimate of the time effect on PGE-M is for a one unit change in time (1 day) on log10 PGE-M. Std.Error = Standard error of the estimate; t value is the t statistic for the estimate. (DOCX) [file pone.0218386.s001.docx]

**Table S1.** **Results from PGE-M multivariable mixed linear model**

| **Variable** | **Estimate** | **Std. Error** | **t value** | **p value** |
| --- | --- | --- | --- | --- |
| Intercept | 2.42E-01 | 2.57E-01 | 0.94 | 3.48E-01 |
| Baseline PGE-M *[Log10(pmol/mg creatinine)]* | 7.76E-01 | 1.50E-01 | 5.17 | 3.09E-04 |
| Sex | -5.22E-03 | 1.13E-01 | -0.05 | 9.64E-01 |
| Race | 1.73E-01 | 8.80E-02 | 1.96 | 7.58E-02 |
| Time [days] | -3.04E-03 | 9.26E-04 | -3.28 | 1.39E-03 |
| Model: PGE-M = Baseline PGE-M + Sex + Race + Time. Estimates are in units of Log10(pmol PGE-M/mg creatinine). The estimate of the time effect on PGE-M is for a one unit change in time (1 day) on log10 PGE-M. Std.Error = Standard error of the estimate; t value is the t statistic for the estimate. | | | | |
