# Supplementary material for: Effects of cessation of cigarette smoking on eicosanoid biomarkers of inflammation and oxidative damage
Source: PLoS One. 2019 Jun 28;14(6):e0218386. doi: 10.1371/journal.pone.0218386 (PMC6599218; doi:10.1371/journal.pone.0218386)
Supplement: S2 Table — Paired t-test employed between baseline and each time-point. Geometric fold change is 10^mean of paired differences). (DOCX) [file pone.0218386.s002.docx]

**Table S2.** **Results from pairwise comparisons of baseline PGE-M vs. each time point**

| **Metabolite** | **Time Point** | **Geometric Fold Change** | **p-value** |
| --- | --- | --- | --- |
| PGE-M | 3 | 0.788 | 0.137 |
| PGE-M | 7 | 0.834 | 0.32 |
| PGE-M | 14 | 0.858 | 0.437 |
| PGE-M | 21 | 0.776 | 0.179 |
| PGE-M | 28 | 0.674 | 0.134 |
| PGE-M | 42 | 0.576 | 0.002 |
| PGE-M | 56 | 0.631 | 0.021 |
| PGE-M | 70 | 0.555 | 0.006 |
| PGE-M | 84 | 0.406 | 0.009 |
