# Supplementary material for: Effects of cessation of cigarette smoking on eicosanoid biomarkers of inflammation and oxidative damage
Source: PLoS One. 2019 Jun 28;14(6):e0218386. doi: 10.1371/journal.pone.0218386 (PMC6599218; doi:10.1371/journal.pone.0218386)
Supplement: S3 Table — Model: 8-iso-PGF 2α = Baseline 8-iso-PGF 2α + Sex + Race + Time. Estimates are in units of Log10 (pmol 8-iso-PGF 2α /mg creatinine). The estimate of the time effect on 8-iso-PGF 2α is for a one unit change in time (1 day) on log10 8-iso-PGF 2α. Std.Error = Standard error of the estimate; t value is the t statistic for the estimate. (DOCX) [file pone.0218386.s003.docx]

**Table S3.** **Results from 8-*iso*-PGF_2α_ multivariable mixed linear model**

| **Variable** | **Estimate** | **Std. Error** | **t. value** | **p. value** |
| --- | --- | --- | --- | --- |
| Intercept | 2.46E-02 | 2.39E-02 | 1.03 | 3.05E-01 |
| Baseline 8-*iso*-PGF_2α_ *[Log10(pmol/mg creatinine)]* | 7.18E-01 | 9.27E-02 | 7.74 | 8.93E-06 |
| Sex | 5.39E-05 | 2.66E-02 | 0.00 | 9.98E-01 |
| Race | -7.39E-02 | 2.64E-02 | -2.80 | 1.73E-02 |
| Time*[days]* | -1.66E-03 | 4.22E-04 | -3.95 | 1.38E-04 |
| Model: 8-*iso*-PGF_2α_ = Baseline 8-*iso*-PGF_2α_ + Sex + Race + Time. Estimates are in units of Log10 (pmol 8-*iso*-PGF_2α_ /mg creatinine). The estimate of the time effect on 8-*iso*-PGF_2α_ is for a one unit change in time (1 day) on log10 8-*iso*-PGF_2α_. Std.Error = Standard error of the estimate; t value is the t statistic for the estimate. | | | | |
