# Supplementary material for: Effects of cessation of cigarette smoking on eicosanoid biomarkers of inflammation and oxidative damage
Source: PLoS One. 2019 Jun 28;14(6):e0218386. doi: 10.1371/journal.pone.0218386 (PMC6599218; doi:10.1371/journal.pone.0218386)
Supplement: S4 Table — Paired t-test employed between baseline and each time-point. Geometric fold change is 10^mean of paired differences). (DOCX) [file pone.0218386.s004.docx]

**Table S4.** **Results from pairwise comparisons of baseline PGE-M vs. each time point**

| **Metabolite** | **Time Point** | **Geometric Fold Change** | **p-value** |
| --- | --- | --- | --- |
| 8-*iso*-PGF_2α_ | 3 | 1 | 0.995 |
| 8-*iso*-PGF_2α_ | 7 | 1.045 | 0.34 |
| 8-*iso*-PGF_2α_ | 14 | 0.872 | 0.163 |
| 8-*iso*-PGF_2α_ | 21 | 0.89 | 0.081 |
| 8-*iso*-PGF_2α_ | 28 | 0.816 | 0.003 |
| 8-*iso*-PGF_2α_ | 42 | 0.775 | 0.011 |
| 8-*iso*-PGF_2α_ | 56 | 0.775 | 0.001 |
| 8-*iso*-PGF_2α_ | 70 | 0.942 | 0.594 |
| 8-*iso*-PGF_2α_ | 84 | 0.689 | 0.007 |
