# Supplementary figures and images for: Effects of cessation of cigarette smoking on eicosanoid biomarkers of inflammation and oxidative damage
Source: PLoS One. 2019 Jun 28;14(6):e0218386. doi: 10.1371/journal.pone.0218386 (PMC6599218; doi:10.1371/journal.pone.0218386)

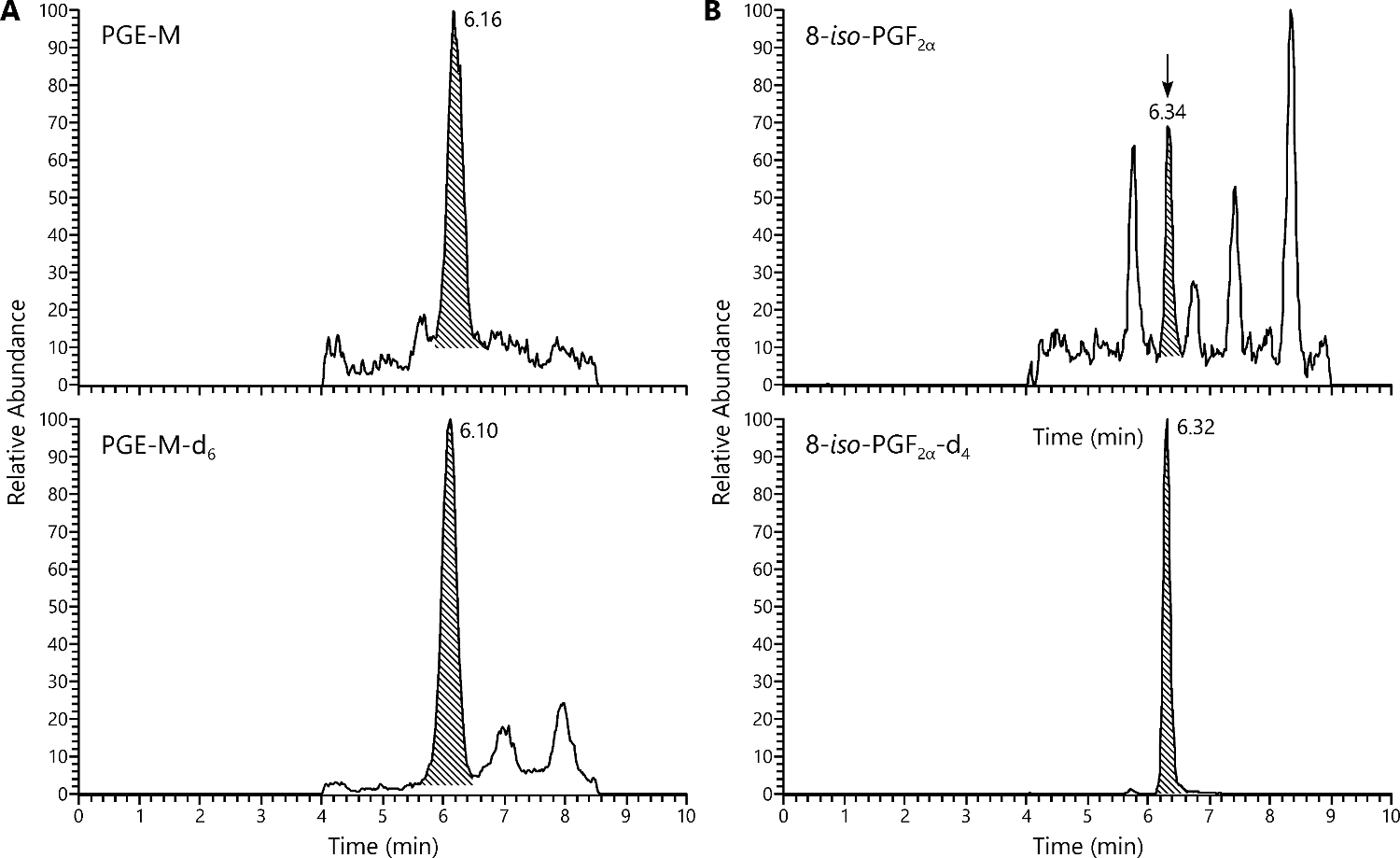

Supplement: S1 Fig — Typical LC-MS/MS trace of PGE-M (A) and 8-iso-PGF2α (B) in a smoker's urine. (TIFF) [file pone.0218386.s005.tiff]
